# Suprarenal Extract in Addison's Disease

**Published:** 1905-09-02

**Authors:** 


### SUPRARENAL EXTRACT IN ADDISON'S DISEASE.

Dr. Gordon Gullan[1] reports two cases of Addison's disease in which treatment by the administration of suprarenal extract was practised. In one, a woman of 32 years, the symptoms had been in existence for two years, and included pigmentation of the skin, attacks of syncope, etc. Suprarenal extract, 5 grains gradually increased to 20 grains, was commenced in August 1904, and was continued until December, when the patient gave birth to a healthy child. Until that date the symptoms had diminished, but on the cessation of the remedy they returned. The suprarenal extract was, therefore, renewed, and has been continued to the present date with great advantage to the health of the patient, but without material modification of the pigmentation. The report insists upon the necessity of full doses of the suprarenal extract—as much as 18 tabloids were given daily—and upon the need for continuous administration. In a second case the suprarenal extract appeared to do good for a time, but the symptoms recurred in an aggravated form and led to a fatal termination. The patient was a young man of 18 years, and the course of the disease was much more acute than in the former instance. Dr. Gullan supports Dr. Byrom Bramwell's view that the cases which improve under suprarenal extract are examples of renal inadequacy, and that cases failing to improve have in addition a lesion of the sympathetic. He thinks that when combined with good diet, fresh air, etc., and commenced at an early stage of the disease, suprarenal extract is of great value and may lead to a permanent cure, though if sclerosis of the adrenals should occur the extract would have to be continued during the rest of the patient's life.

Sir Lauder Brunton[2] has also written on the value of suprarenal extract, and says that under its influence cases of Addison's disease "begin to show either amendment or perhaps, occasionally, complete recovery."

Osler quotes Kinnicutt's statistics which show that out of 48 cases of the disease treated with suprarenal extract 6 were cured and 22 improved.

Dr. C. O. Hawthorne[3] has described a patient—a woman of 48 years—who 11 years previously had been recognised as suffering from Addison's disease with marked pigmentation of the skin and buccal mucous membrane. She remained for several years in poor health, but the dark patches on the skin gradually disappeared, and there now remains no evidence of undue pigmentation except several slate-coloured patches on the deep surfaces of the lips and cheeks. Under suprarenal extract the patient announced much improvement in her general condition, and more particularly the cessation of attacks of vomiting which previously had been very troublesome. Whilst it must be allowed that suprarenal extract in Addison's disease cannot boast the therapeutic certainty which attends the prescription of thyroid extract in myxœdema, it is still true that in a percentage of cases it produces good results, and these would probably be more marked were the disease diagnosed in its earliest stages and the remedy given continuously and in full doses.

[1] Lancet, Aug. 19, 1905. [2] "The Action of Medicines," 1897, p. 52. [3] Polyclinic Jour., July 1904.

